# Periodic Mesoporous Organosilica Nanocubes with Ultrahigh Surface Areas for Efficient CO_2_ Adsorption

**DOI:** 10.1038/srep20769

**Published:** 2016-02-12

**Authors:** Yong Wei, Xiaomin Li, Renyuan Zhang, Yong Liu, Wenxing Wang, Yun Ling, Ahmed Mohamed El-Toni, Dongyuan Zhao

**Affiliations:** 1 Department of Chemistry, Collaborative Innovation Center of Chemistry for Energy Materials (iChEM), Laboratory of Advanced Materials, Shanghai Key Laboratory of Molecular Catalysis and Innovative Materials, Fudan University, Shanghai 200433, P. R. China; 2 School of Materials Science and Engineering, Key Laboratory of Advanced Civil Engineering Materials of Ministry of Education, Tongji University, 4800 Caoan Road, Shanghai, 201804, P. R. China; 3 King Abdullah Institute for Nanotechnology, King Saud University, Riyadh 11451, Saudi Arabia; 4 Central Metallurgical Research and Development Institute, CMRDI, Helwan 11421, Cairo, Egypt

## Abstract

Ultrahigh surface area single-crystals of periodic mesoporous organosilica (PMOs) with uniform cubic or truncated-cubic morphology and organic/inorganic components homogeneously distributed over the whole frameworks have successfully been prepared by a sol-gel surfactant-templating method. By tuning the porous feature and polymerization degree, the surface areas of the obtained PMO nanocubes can reach as high as 2370 m^2^/g, which is the highest for silica-based mesoporous materials. The ultrahigh surface area of the obtained PMO single crystals is mainly resulted from abundant micropores in the mesoporous frameworks. Furthermore, the diameter of the nanocubes can also be well controlled from 150 to 600 nm. The materials show ultrahigh CO_2_ adsorption capacity (up to 1.42 mmol/g at 273 K) which is much higher than other porous silica materials and comparable to some carbonaceous materials. The adsorption of CO_2_ into the PMO nanocubes is mainly in physical interaction, therefore the adsorption-desorption process is highly reversible and the adsorption capacity is much dependent on the surface area of the materials. Moreover, the selectivity is also very high (~11 times to N_2_) towards CO_2_ adsorption.

Due to the highlighting characteristics of well-defined ordered mesostructure, controlled pore size, large surface area and pore volume, varieties of the frameworks, and easy functionality, the mesoporous silica materials have attracted a great deal of attention, and have been proposed as potential platforms in various fields, such as molecular imprinting, drug delivery systems, biosensors, bio-imaging, optical devices, energy storage, low-*k* dielectric layers, nanocatalysis, and adsorbents for pollutes from both air and aqueous solutions^1^,^2^,^3^,^4^,^5^,^6^,^7^,^8^,^9^. As one of the most important members of silica-based mesoporous materials, periodic mesoporous organosilicas (PMOs)^10^,^11^,^12^ are the most representative organic–inorganic hybrid porous materials with the organic/inorganic components (silsesquioxane, O_1.5_Si-R-SiO_1.5_) completely and homogeneously distributed over the whole frameworks at the molecular level. By introducing adequate organic R parts homogeneously within the mesoporous frameworks, PMOs can be endowed with a variety of features^13^,^14^,^15^ such as tunable biodegradation, improved biocompatibility, varied hydrophobicity, hydrophilicity and amphiphilicity, versatile functionality, *etc*. Although the surface areas of PMOs are normally higher than the other silica-based mesoporous materials, the highest surface area (1880 m^2^/g)^16^ for PMOs reported previously is still much lower than other kinds of porous materials^17^, such as microporous carbon (2000~5000 m^2^/g)^18^,^19^ and metal/covalent-organic frameworks (MOF/COF) (3000~7000 m^2^/g)^20^,^21^,^22^, *etc*. If the surface area of PMO materials can be improved close to that of activated carbon and MOF, this novel family of molecularly organic-inorganic hybrid nanomaterials with tunable compositions would be produced to substantially extend the application potentials of PMOs, especially in heterogeneous catalysis, pollutes adsorption and gas capture.

Among all the gaseous pollutes, CO_2_ capture and separation is in urgent demand, because CO_2_ is the predominant greenhouse gas as well as its separation from natural gas and syngas is indeed very important. Until now, many kinds of new materials have been promoted as the promising adsorbents, including physical absorbents, functional microporous or mesoporous materials, carbonaceous materials, MOFs, and chemical-looping combustion using metal oxides, hydroxides and carbonates^23^,^24^. It has been demonstrated that CO_2_ adsorption capacity is greatly dependent on both the physical adsorption (Van Der Waals interaction) and chemical sorption (the affinity interaction between CO_2_ and surface functional groups)^25^,^26^. So CO_2_ adsorption capacity is quite related to the accessible surface areas and the number of functional groups in adsorbents. By means of high stability and easy functionalization, PMO materials could be one of the most important CO_2_ adsorbents, if the surface area could well-matched with microporous carbons and MOFs.

Herein, ultrahigh surface area PMO single-crystals (mesostructure symmetry of group space Pm-3n) with cubic or truncated-cubic morphology and organic/inorganic components homogeneously distributed over the whole frameworks have successfully been prepared by a sol-gel method combined with surfactant-templating process. The diameter of the PMO nanomaterials can be well controlled from 150 to 600 nm. The obtained uniform PMO nanocubes exhibit ultrahigh surface areas especially that of the nanomaterial with a size of 200 nm reach up to 2370 m^2^/g, which is the highest for silica-based mesoporous materials, very close to that of some MOF and carbonaceous materials. It is found that the origin of the ultrahigh surface area is mainly resulted from micropores constructed by the silanol groups in the mesoporous organosilica frameworks. The PMO materials with ultrahigh surface areas can be used as CO_2_ adsorbents, showing much high capacity (up to 1.42 mmol/g at 273 K and 0.97 mmol/g at 298 K) and excellent selectivity (11 times to N_2_ at 273 K). Since the adsorption of CO_2_ on the PMO nanocubes is mainly based on physical interaction, the capacity is linearly dependent on the surface area and the adsorption-desorption process is highly reversible.

## Results and Discussion

**Figure 1:**
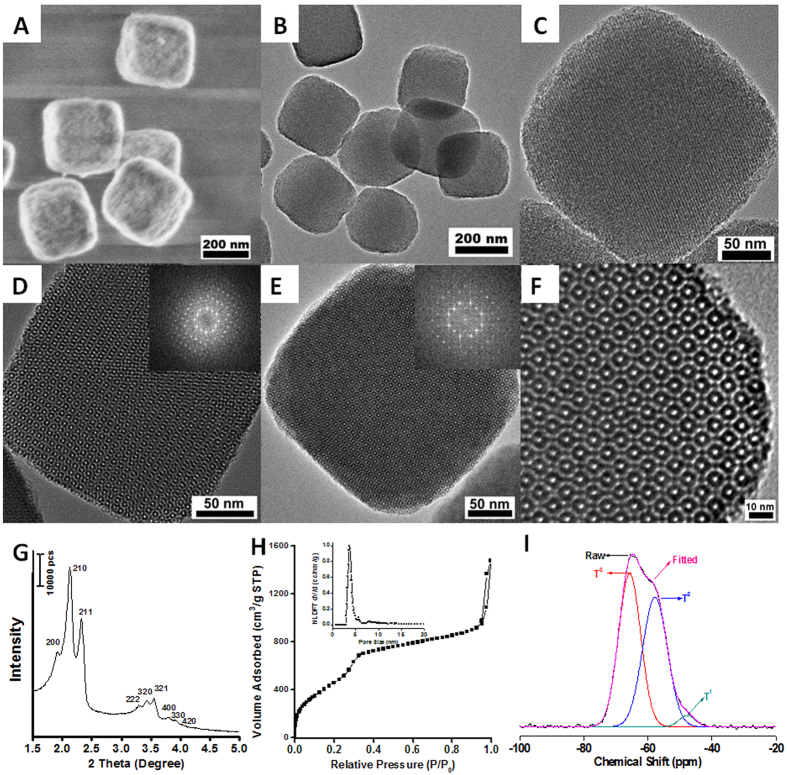
The characterization of the typical PMO nanocubes with a size of 200 nm. FESEM (**A**), TEM (**B–F**) images, small-angle XRD patterns (**G**), nitrogen adsorption-desorption isotherms (**H**) and the corresponding NLDFT pore size distribution plots (inset **H**), ^29^Si MAS NMR spectra (**I**). The HRTEM images (**C–E**) are viewed from (210), (110) and (100) planes, respectively. Insets (**C,D**) and (**E**) are the corresponding fast Fourier transform (FFT) of the TEM images.

The ethane-bridged PMO mesoporous single-crystals^32^,^33^,^34^ can be synthesized *via* a simple sol-gel method at room temperature, by using surfactant tetradecyltrimethylammonium chloride (TTAC) as a template, bis-triethanoxylsilylethane (BTSE) as a silica precursor and ammonia as the catalyst, and the synthesis recipes for the PMO nanomaterials with different sizes are listed in Table S1. The PMO nanocubes with the size of 200 nm (sample 2) was synthesized with molar ratio of 1/0.4/10.5/2470 (TTAC/BTSE/NH_3_/H_2_O). Typically, 0.4 g of TTAC and 1.5 mL of ammonia solution (28 wt%) were added into 60 ml of water and stirred for 30 min at ~700 rpm, before the addition of 0.1 mL of BTSE. After 20 h, the as-prepared samples were collected and extracted, then characterized with several methods. The HRSEM image (Fig. 1A) of the represent crystals with the diameter of ~200 nm shows that the crystals exhibit discrete and highly monodispered cubic morphology with six equivalent {100} faces. The TEM images (Fig. 1B–F) of the obtained PMO nanocubes along <210>, <110>, <100> directions and corresponding fast Fourier transform (FFT) images reveal well regular periodicity and less defect at mesoscale level. Nine well-resolved diffraction peaks can be observed in the small-angle X-ray diffraction (SA-XRD) patterns (Fig. 1G) of the PMO single-crystal nanocubes after the removal of the surfactant templates by solvent extraction of ethanol, clearly revealing the highly ordered mesostructure. The three well-resolved diffraction peaks at 1.9~2.3° are indexed as the 200, 210 and 211 reflections of simple cubic mesostructure with space group of Pm-3n^35^, suggesting a mesoscale ordering of the nanocubes in cubic symmetry. These apparent peaks shown in the range of 3.3~4.5° can well be indexed as the 222, 320, 321, 400, 330, 420 diffractions, and the lattice parameter (*a*) is calculated to be 9.21 nm.

The nitrogen sorption isotherms of the obtained mesoporous PMO nanocubes (Fig. 1H) show that the volume of adsorbed N_2 _raises rapidly at relative pressure (P/P_0_) less than 0.05 and increases steadily in the range of P/P_0_ at 0.05~0.35, indicating that the single crystals contain abundant micropores and small mesopores. Moreover, in the range of P/P_0_ larger than 0.95, the volume of adsorbed liquid N_2_ also increases sharply, which is contributed to the nanoparticle accumulation, implying the highly uniform particle sizes of the nanocubes. The Brunauer–Emmett–Teller (BET) specific surface area of the PMO nanocubes with a size of 200 nm can reach as high as 2370 m^2^/g and the total pore volume up to 1.98 cm^3^/g. Since the high surface area of the PMO materials usually ranges from 1390 to 1880 m^2^/g (Table S2), the PMO nanocubes has the highest value of specific surface area among all the mesoporous organosilica materials yet reported. The microporous surface area and micropore volume are calculated to be 1760 m^2^/g and 0.51 cm^3^/g by the *V*-*t* method, occupying nearly 70% area but only 1/4 volume in the total surface area and total pore volume. The pore size distributions (Fig. 1H inset) are plotted based on non-local density functional theory (NLDFT) method^36^ with cylinder/slit-pore model, and the mesopores are centered at 3.7 and 5.7 nm with bimodal distributions. The bimodal mesopore size distributions of the ethane-bridged PMO single crystals are well in accord with the A_3_B type *Pm-3n* structure as the three-dimensional (3D) model reported previously^35^ (Fig. S1) (left: a unit cell, right: the arrangement of two types of cages).

The chemical composition of the mesoporous PMO nanocubes was verified with solid-state NMR spectroscopy. The resonance with chemical shift at about 5.0 ppm in the ^13^C CP NMR spectrum (Fig. S2) of the mesoporous PMO nanocubes can be attributed to C species of the bridged ethane moiety (–CH_2_CH_2_–) originated from organosilane precursors. The bands at 58.0, 39.6 and 15.9 ppm are caused by the residues surfactants and the ethoxyl groups formed during the extraction of the surfactants. ^29^Si MAS NMR spectra (Fig. 1I) of the PMO nanocubes with 250 nm in size show a broad band in the range of –80 to –40 ppm, revealing the existence of Si atoms bridged by the ethane groups (T^n^ sites). To investigate the species of T^n^ sites, the broad band can be simulated as three peaks centered at –65.7, –57.9 and –47.6 ppm, which can be assigned as T^3^ [SiC(OSi)_3_], T^2^ [(HO)SiC(OSi)_2_] and T^1^ [(HO)_2_SiC(OSi)] sites, respectively. The percent ratio of the normalized peak areas for T^3^, T^2^, T^1^ is calculated to be 48.6%, 48.7%, 2.7%, respectively, and the corresponding condensation degree is about 82.0%, which is much lower than that in most reports about ethane-bridged mesoporous silica. Nearly half of Si atoms are connected to hydroxyl groups, these silanol groups provide great amount of micropores and lead to the increase of specific surface area. Thus, the lower framework density, ordered cubic mesostructure and lower polymerization degree might result in the ultrahigh surface area of the PMO nanocubes.

**Figure 2:**
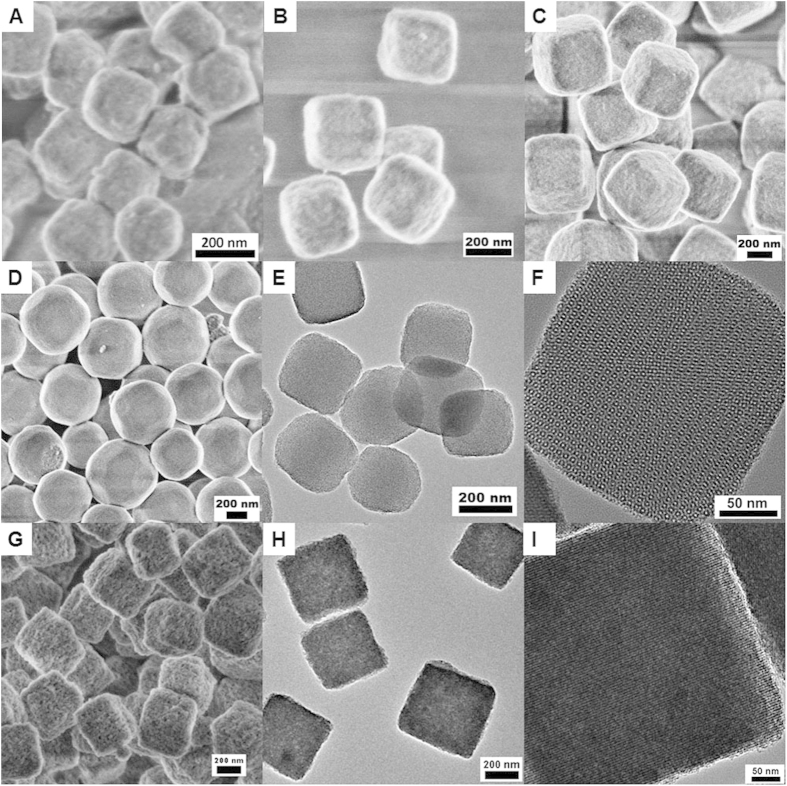
The characterization of the PMO nanocubes with electron microscopy. FESEM images (**A–D**) of the PMO nanocubes and truncated-cubes with sizes of 150 nm (**A**), 250 nm (**B**), 400 nm (**C**) and 600 nm (**D**); TEM images for the PMO nanocubes with a size of 250 nm (**E,F**); HRSEM (**G**) and TEM (**H,I**) images of the PMO nanocubes with a size of 250 nm after hydrothermal treatment at 120 °C.

**Figure 3:**
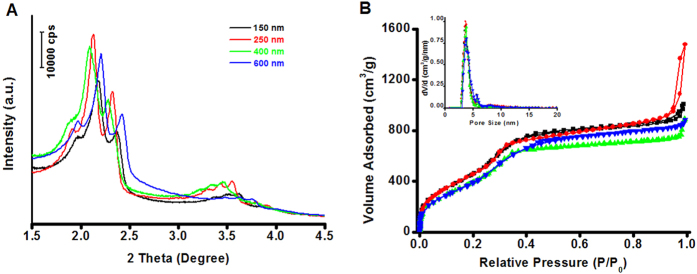
The characterization of the PMO nanocubes with different sizes. Small-angle XRD patterns (**A**), nitrogen adsorption–desorption isotherms (**B**) and the corresponding NLDFT pore size distributions (inset **B**) of the extracted ethane-bridged PMO materials with different particle sizes of 150 nm (black), 200 nm (red), 400 nm (green) and 600 nm (blue).

As aforementioned, the surface area of the PMO materials is affected by the polymerization degree of the materials, which is relied on the synthesis condition such as the alkalinity and the ratio of reactants. In order to verify the relationship between the high surface area of the PMO materials and the polymerization degree, the amount of surfactant and ammonia were adjusted (Table S1). PMO crystals with different polymerization degree and particle sizes in a wide sub-micrometer range could be obtained while maintaining the ordered mesostructure and uniform external crystal-like morphology. For example, as the ammonia amount gradually increased, the particle sizes of the nanocubes varied from 150 to 400 nm (Fig. 2A–C), and the surface of the cubes with smaller sizes (150, 200 and 250 nm) is slightly bumpy because of the mesoporous defects. As shown in the SEM and TEM images, the obtained PMO nanocubes (samples 1, 3, 4) with different sizes such as 150, 250 and 400 nm possess well-defined cubic morphology as well as uniform particle sizes and highly ordered cubic mesostructure (Fig. 2E,F, S3, S4). At a low TTAC/BTSE ratio, the particles (samples 5, 6, 7) have truncated-cubic morphology, and the particle size can be also tuned in the range of 300~600 nm (Fig. S5). The PMO truncated-cubes are also monodispersed and discrete (Fig. 2D), with 26 faces (Fig. S6A) in three types, and also have highly ordered mesostructure as shown in the HRTEM image (Fig. S6B–D)^34^. All the SA-XRD patterns of the PMO nanomaterials (Fig. 3A) show six well-resolved diffraction peaks in the 2θ range of 1.9~3.6°, which further confirm the highly ordered cubic mesostructure. The nitrogen isotherms (Fig. 3B) of the PMO nanocubes and truncated-cubes also show type IV curves for uniform mesopores. The BET specific surface areas of the mesoporous PMO nanocubes with the size of 150, 250, 400 nm and truncated-cubes with 600 nm in size are calculated as 2220, 1960, 1670 and 1570 m^2^/g respectively, and these materials have similar pore size distributions (Fig. 3B inset) with small mesopores centered at about 3.7 or 3.8 nm and large mesopores at ~5.7 nm (Table 1).

Many factors could affect the surface area of materials. For example, the particle size influences the external surface area of solids, the structure parameters of the mesostructure change the mesopore and micropore surface areas impacted by the polymerization degree. In order to reveal the dominating factor for the surface area, we estimated the external and mesopore surface area of the PMO nanocubes based on the equations (See Experimental Section). The outer surface areas of the PMO nanomaterials with the size of 150, 200 and 250 nm are calculated to be 17.2, 16.1 and 11.3 m^2^/g, respectively (Table 2), while that for the PMo nanomaterials with the size of 400 (6.1 m^2^/g) and 600 nm (4.3 m^2^/g) with relatively larger sizes are much lower. All the values are much smaller compared to the total surface area, so that the influence of the size on the surface area is usually ignored. The mesoporous porosity (*P*_*me*_) of the PMO materials was also estimated (Table 2), and the values were compared with the total porosity (*P*_*t*_). It is observed that the ratio of mesopore porosity to total porosity (*P*_*me*_*/P*_*t*_) is quite close to that of mesopore volume to the volume of complementary and primary pores (*V*_*me*_*/V*_*P*_) with very little difference below 5% (Table 2), indicating that the suppose and calculations are proper. Furthermore, the mesopore surface areas (512~780 m^2^/g) were estimated very close to the measured one (*S*_*me*_, 470~663 m^2^/g), *i.e.*, the mesopore surface area (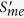) of sample 3 is estimated to be ~ 626 m^2^/g, with a deviation of only 5.5% compared to the experimental value (663 m^2^/g). It is found that the mesoporous surface areas of all the PMO nanomaterials are no more than one third of the total surface areas, so that the microporous surface areas (*S*_*mic*_, 1030~1750 m^2^/g) play the dominant role in the total surface areas, and the factor that affect the micropore surface areas should also be the key element among all the cases which influence the total surface area of PMO materials. For the PMO cubes with the diameter of 150 and 200 nm, they are both synthesized at quite lower ammonia concentrations than other materials, and possess very high micropore surface areas (1750 and 1760 m^2^/g) which were very close to each other, so that they have much higher surface areas. However, the sample for the PMO cubes with the diameter of 200 nm has higher surface areas than another one because of its relatively higher mesopore surface areas (610 vs 470 m^2^/g). This is because the one with the diameter of 200 nm has much more perfect mesoporous crystals, which could be evidenced by the much more well-dissolved peaks of the sample (Figs 1G and 3A) and the defects (Fig. 2A) on the surfaces of the 150 nm one. Thus, for the materials with similar micropore surface areas, the ordered mesostructure would also help to enhance the specific surface area of the materials.

**Figure 4:**
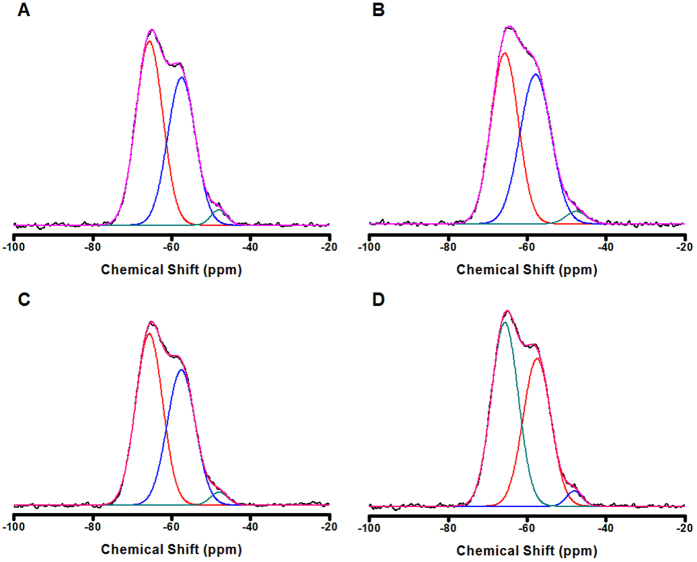
^29^Si MAS-NMR spectra of the PMO nanocubes with different size. (**A**) 150, (**B**) 250, (**C**) 400, (**D**) 600 nm. Black lines show the raw data of the materials, while the red, blue, cyan, purple ones present the T^3^, T^2^, T^1^ bands and fitted data, respectively.

In silica-based materials, the micropores are mainly formed between the ≡Si-OH groups, or =Si(-C)-OH groups for PMOs. The polymerization degree of the materials is decided by the amounts of =Si(-C)-OH groups in mesoporous organosilica, which greatly influences the micropore surface area of the materials. Under basic conditions, the hydrolysis rate of silica precursors is very high while their condensation process is delayed, and usually the polymerization degree is a little larger at high alkalinity or high base concentrations. ^29^Si MAS-NMR spectra (Fig. 4) for the PMO nanocubes and truncated-cubes were studied, the contents of T^3^, T^2^, T^1^ species and the polymerization degree of the PMO nanomaterials are listed in Table 3. All the materials have very high T^2^ contents and possess ultralow polymerization degree compared to usual silica-based materials, thus they all have very high surface areas (>1570 m^2^/g). Not accidently, the PMO nanomaterials with the size of 400 and 600 nm have slightly higher condensation degree since the ammonia concentration in their synthesis procedure is higher than the others. The PMO nanocubes with 200 nm in size and the lowest polymerization degree bear the highest surface area (~2370 m^2^/g), and the truncated-cubes with the size of 600 nm have the lowest surface area of ~1570 m^2^/g since the latter has the highest condensation degree of 83.7%. From these results, we can conclude that the surface area of PMO materials is highly related to the condensation degree and the control of the condensation degree is an effective method to obtain porous silica with ultrahigh surface area[Table t4].

In order to further investigate the relationship between the polymerization degree and the pore surface areas, hydrothermal treatments of the PMO nanocubes with 250 nm in size were performed at 60, 80, 100 and 120 °C for 24 h, respectively (named as HT-t, t is the hydrothermal temperature). The SA-XRD patterns (Fig. S7) of the samples are quite similar to the untreated nanocubes, revealing that the ordered mesostructure is maintained after hydrothermal treatments. The diffraction peaks in the range of 3.0~3.5° clearly indicate that the mesostructure is further improved for HT-60 and HT-80 while that is partially destroyed for HT-100 and HT-120 during the hydrothermal treatment. Due to the reorganization of the silica during the hydrothermal treatment, the secondary mesopores on the surface of the nanocubes are formed, which can be clearly observed from the SEM images of treated samples (Fig. 2G and Fig. S8). The TEM images (Fig. 2H,I) of the HT-120 sample also indicate that it has much more pores than the untreated nanocubes, the lower contrast in the cube further confirms the formation of secondary mesopores and the partial destroy of the ordered mesostructure. The rearrangement of the silica framework can further lead to the increase of the polymerization degree of silica, which is evidenced by ^29^Si-MAS NMR (Fig. S9). As the hydrothermal temperature increases, the content of T^3^ species in the total silica increases, changes from 50.0% for the untreated nanocubes to 68.7% for the treated nanocubes treated at 120 °C, while that of T^2^ and T^1^ species gradually decreases. When the temperature is higher than 100 °C, the content of T^1^ species is less than 1% and T^2^ species decrease to ~30%, and the condensation degree increases up to about 90% (Table S3). Correspondingly, nitrogen sorption isotherms (Fig. S10A) show that the BET specific surface areas gradually decreases from 1960 m^2^/g for the original PMO nanocubes to 1500 m^2^/g (HT-60), 1120 m^2^/g (HT-80), 940 m^2^/g (HT-100) and finally only 460 m^2^/g for HT-120 (Table S4). The corresponding pore size distribution plots (Fig. S10B) show that the secondary mesopore size after the hydrothermal treatment is about 8.1 nm. At the same time, the micropore percentage of the original nanocubes gradually decrease as the temperature increases. While the mesopore surface areas little change, the result is quite consistent with that reported in the literature^37^.

Before the treatments, the highly porous nanocubes have ordered mesopores and great amount of micropores in the pore walls. There are abundant =Si(-C)-OH groups on both microporous and mesoporous surfaces. During the hydrothermal process, most of =Si(-C)-OH groups on the surface are turned into =Si(-C)-O-Si(-C)= bonds, the polymerization degree of the mesoporous organosilica materials increases correspondingly. After the hydrothermal treatment at 120 °C, the micropores totally disappear while only about one third of =Si(-C)-OH groups in the untreated pristine nanocubes condenses into =Si(-C)-O-Si(-C)= groups. The micropores may be transformed in two routes, partially integrated into the mesopores because of the framework shrinkage during the thermal process, else are sealed up by the =Si(-C)-O-Si(-C)= groups as well as the arrangement of silica frameworks and become inaccessible. Thus the residual =Si(-C)-OH groups are mostly on the surface of mesopores and a small proportion in the inaccessible closed micropores in the pore walls. For mesoporous silica-based materials with a high surface area, the micropore surface area is the most important constituent part of the total surface area. Most of these materials were synthesized at a low temperature (normally <60 °C) with ammonia as the catalyst, such as the PMO microspheres with super-microporous feature and highest surface area of 1880 m^2^/g before the present work^16^. While, the silica materials synthesized at the high temperatures always have a high polymerization degree and therefore have lower surface areas, as well as the materials post-treated at the high temperatures which is also evidenced by the results shown in the present paper. As demonstrated here, the micropores are the slits constructed by the silanol groups, and the number of the silanol groups greatly affects the micropore surface area of the silica-based materials.

**Figure 5:**
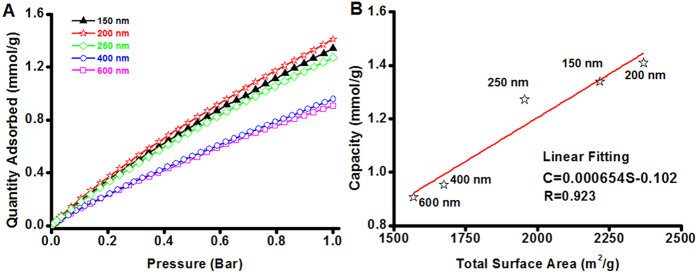
The CO_2_ adsorption performance of the ethane-bridged PMO nanocubes. (**A**) The CO_2_ adsorption curves of the PMO nanocubes and truncated-cubes with sizes of 150, 200, 250, 400, 600 nm and (**B**) the relationship between CO_2_ capacity and total surface area.

The CO_2_ adsorption performance of the PMO nanocubes and truncated-cubes with ultrahigh surface areas and uniform particle sizes of 150, 200, 250, 400 and 600 nm was investigated. All the isotherms (Fig. 5A) are linearly coefficient, which is quite different from the CO_2_ adsorption performance on functional adsorbents (usually type I isotherms)^26^. At 273 K, CO_2_ adsorption capacity of the PMO nanocubes with a size of 200 nm can reach up to 1.42 mmol/g, and the capacities for the PMO nanocubes with the sizes of 150, 250, 400 nm and PMO truncated-cubes with 600 nm in size are calculated to be 1.34, 1.27, 0.95, 0.90 mmol/g, respectively. The results suggest that the absorption capacity is quite related to the BET surface area of the materials (Fig. 5B), and 0.654 μM of CO_2_ adsorbed on per m^2^ of PMO surface based on the fitted linear plot. On the other side, the linear fitting of the relationship between the micropore surface area and the CO_2_ adsorption capacity is found to be not proper (R is only 0.742, Fig. S11). So that the adsorption of CO_2_ onto the surface of the materials is not site-selective, both the mesopore and micropore surfaces attribute to the adsorption process. The CO_2_ adsorption-desorption process on PMO materials is highly reversible (Fig. S12). Even at higher temperature of 298 K, the CO_2_ adsorption capacity of the PMO nanocubes with 200 nm in size and highest surface area (2370 m^2^/g) can still reach up to 0.97 mmol/g (Fig. S13). Correspondingly, the isosteric heat of CO_2_ adsorption (Q_is_) on the PMO nanocubes was calculated using the Clauysius-Clapeyron equation (Eq. 7) and the adsorption data at different temperatures (Fig. 5A and S13), and the relationship between the values of Q_is_ and CO_2_ adsorption capacities are plotted in Fig. S14. At the lowest capacity of 0.02 mmol/g for CO_2_ adsorption, the Q_is_ value is 12.80 kJ/mol, while at the capacity of ~0.2 mmol/g, the value increases to 14.29 kJ/mol. And as the CO_2_ capacity varies in the range of 0.25 to 0.97 mmol/g, the heat of adsorption varies very little (10.04~10.80 kJ/mol), all these values are quite smaller than that of most chemical adsorbents (>30 KJ/mol)^38^. For the ethane-bridged PMO nanocubes, the bridged groups (-CH_2_-CH_2_-) and the surface silanol groups only have very low chemical interaction with CO_2_ so that the adsorption behavior is mainly caused by Van Der Waals force, and therefore the adsorbed amount present a linear relation versus the equilibrium pressure. As discussed above, the CO_2_ adsorbed onto the surface of the PMO nanocubes by physical interaction, meaning that the adsorption capacity is highly relied on the surface area of the materials. Therefore, for the PMO nanocubes and truncated-cubes prepared by using TTAC as a template, higher surface area of the materials leads to the larger capacity towards CO_2_ adsorption. The CO_2_ adsorption performance on several silica-based adsorbents is summarized in Table 4. It can be seen that the PMO nanocubes in the current work show higher capacity than not only the non-functional porous silica materials^40^,^41^,^43^,^44^ and also a large proportion of the amine-functional silicas^39^,^41^,^42^,^43^,^44^. Moreover, the PMO nanocubes also show very high selectivity, since the adsorption capacity for N_2_ (Fig. S15) at 273 K is only about 0.13 mmol/g, which is one eleventh of CO_2_.

## Conclusions

In summary, ethane-bridged PMO single crystals with uniform cubic and truncated-cubic morphology have successfully been synthesized by a surfactant-templating sol-gel method, *via* self-assembly of TTAC/BTSE in aqueous ammonia solution. The PMO crystals have highly ordered mesostructure, highly microporous feature (about 70% in total surface area), monodispered particles with tunable uniform sizes (100~600 nm), ultrahigh surface areas (up to 2370 m^2^/g) and similar bimodal mesopore sizes (~3.7 and~5.7 nm). This is a big progress in attempting the extreme of the surface area of mesoporous silica, the obtained mesoporous organosilica based materials have the highest surface area yet reported, which is comparable to activated carbon and some other materials. It is found that the increase of microporous surface area plays a key role to achieve high surface area for the porous organosilica and the surface areas is highly relied on the polymerization degree of the materials. The PMO materials are used as CO_2_ adsorbents and show excellent selectivity towards N_2 _and very high capacity up to ~1.42 mmol/g with very low isosteric heats (10.04~14.29 KJ/mol). Moreover, the CO_2_ adsorption capacity is mainly depend on the surface area of the materials, and the adsorption-desorption process is highly reversible. With ordered mesoporous structure, relatively smaller particle sizes, and the easily-functionalized organic groups embedded in the pore walls, the PMO single crystals may find new applications in many other fields, such as catalysis, separation, bio-imaging and drug delivery systems.

## Experimental Section

### Materials Preparation

Tetradecyltrimethylammonium chloride (TTAC) was purchased from TCI and bis-triethanoxylsilylethane (BTSE) from Meryer, other reagents were purchased from Guoyao Chemical Company. All reagents were used as received. The ethane-bridged PMO single crystals were synthesized by a surfantant-templating sol-gel method in ammonia solution with TTAC as the structure-directing agent at room temperature. The reactant compositions for the synthesis of TTAC templated ethane-bridged PMO materials were listed in Table S1. Typically, the PMO nanocubes with a size of 200 or 250 nm were synthesized as followed, 0.4 g of TTAC and 1.5 mL (or 2 mL) of ammonia solution (28 wt% aqueous solution) were added into 60 ml of deionized water and stirred for 30 min (a rate at ~700 rpm) before the addition of 0.1 mL of BTSE. The as-prepared samples were collected by centrifugation for 20 h, and later on extracted with 3:97 HCl/ethanol at 60 °C for 6~12 h for twice prior to characterizations. The 250-nm PMO nanocubes were also hydrothermal treated at 60 to 120 °C for 24 h before the separation.

### Characterization

The mesostructure of the samples was characterized using powder small-angle X-ray diffraction (SAXRD, Bruker D8 X-ray diffractometer with Ni-filtered Cu-K*α* radiation) at 40 kV. Field-emission scanning electron microscopy (FESEM) images were obtained with a Hitachi S4800 microscope and transmission electron microscopy (TEM) measurements conducted on a JEOL 2011 microscope operated at 200 kV. The nitrogen sorption isotherms were measured at 77 K with a Quanta chrome Autosorb-1MP analyzer. Before the measurements, the samples were firstly outgassed in vacuum at 120 °C for at least 12 h. The specific surface areas were calculated by the multipoint Brunauer–Emmett–Teller (BET) method with P/P_0_ in the range of 0.05~0.25 with R >0.999 and c >20. The pore size distributions were plotted based on non-local density functional theory (NLDFT) method with cylinder/slit-pore mode or using the Barrett-Joyner-Halenda (BJH) model from the adsorption branch isotherms, the total pore volumes were estimated from the amount of nitrogen adsorbed at the highest relative pressure of P/P_0_ at about 0.995. The micropore surface areas and volumes were calculated by the *V*-*t* plots with the best fitting coefficient (R >0.999) with *P*/*P*_0_ in the range of 0.05–0.6. CO_2_ adsorption was performed on a Micromeritics ASAP-2020 analyzer at certain temperature (298,288 and 273 K). ^29^Si CP-MAS NMR measurements were performed on a Bruker AVANCE III 400 WB spectrometer. The spinning rate was 12 kHz and a total number of 20,000 scans were recorded with 6 s recycle delay for each sample. ^13^C-MAS NMR measurements were performed on Varian Infinity Plus 400 NMR spectrometer. The spinning rate was 4 kHz and a total number of 800 scans were recorded with 4 s recycle delay for each sample.

### Calculations

The external surface area can be calculated following equation (1).


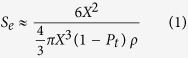


In Eq. (1), the opened pores on the outer surface are not excluded, X is the size of nanocubes and the symbol ρ denotes the framework density of the material, in the case of ethane-bridged organosilica, *ρ* = 1.6 cm^3^/g^45^ was mostly used. *P*_*t*_ is the total porosity calculated as followed:


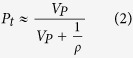


*V*_*P*_in Eq. (2) is the volume of complementary. The primary pores was evaluated by excluding the secondary and textural mesopores from the total pore volume, and calculated based on Eq. (3) in two routes, in which *V*_*t*_, *V*_*me*_, *V*_*mic*_ and *V*_*in*_ are the total, mesoporous, microporous and inter-particle volume of the samples, respectively.


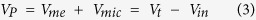


The estimated mesoporous porosity (*P*_*me*_) and surface area (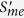) were estimated based on Eq. (4) and (5).


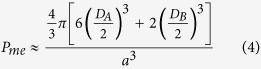



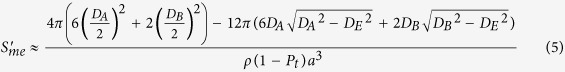


*D*_*A*_ and *D*_*B*_, are the diameters of A-cages and B-cages defined at the maximum of the pore size distributions calculated by NLDFT method as listed in Table 1, *D*_*E*_ is the entrance size of the cage-like pores, which was estimated to be 1.6 nm in all cases. The unit cell parameter (a) was calculated based on equation (6), where *d*_210_ is the d-spacing along <210> axis calculated based on Bragg equation.


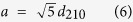


The unit cell parameter, *d*_210_ and the total porosity *P*_*t*_ have also been listed in Table 1, and the other parameters (*S*_*e*_, *P*_*me*_, 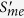) in Table 2.

The isosteric heat of CO_2_ adsorption (Q_is_) on periodic mesoporous organosilica materials, calculated using the Clauysius Clapeyron equation (Eq. 7) and the adsorption data at different temperatures.


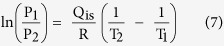


## Additional Information

**How to cite this article**: Wei, Y. *et al.* Periodic Mesoporous Organosilica Nanocubes with Ultrahigh Surface Areas for Efficient CO_2_ Adsorption. *Sci. Rep.* **6**, 20769; doi: 10.1038/srep20769 (2016).

## Supplementary Material

Supplementary Information

## Figures and Tables

**Table 1:** Adsorption and structural parameters for the ethane-PMO materials prepared by using surfactant-templating sol-gel method.

PMO Sample	Size (nm)	Pore Volume (cm^3^/g)				*a*(nm)	Pore Size (nm)	Surface Area (m^2^/g)		
		V_t_	V_mic_	V_me_	V_inter_			S_BET_	S_mic_	S_me_
1	150	1.56	0.28	0.90	0.38	9.09	3.8/5.7	2220	1750	470
2	200	1.98	0.51	1.11	0.36	9.21	3.8/5.7	2370	1760	610
3	250	2.29	0.49	0.86	0.94	9.27	3.7/5.7	1960	1290	660
4	400	1.38	0.34	0.73	0.31	9.43	3.7/5.7	1670	1210	460
5	600	1.37	0.43	0.75	0.19	8.94	3.7/5.7	1570	1030	540

**Table 2:** Estimation of the external and mesopore surface areas of the PMO materials.

Sample	Size (nm)	P_t_ (%)	P_me_ (%)	P_me_/P_t_	V_me_/V_p_	S_e_ (m^2^/g)	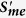 (m^2^/g)
1	150	0.653	0.481	0.737	0.764	17.2	649
2	200	0.722	0.463	0.641	0.685	16.1	780
3	250	0.683	0.434	0.635	0.638	11.3	626
4	400	0.632	0.412	0.652	0.682	6.1	512
5	600	0.652	0.484	0.742	0.635	4.3	638

**Table 3:** The content of different silica species and polymerization degree (D) of the extracted ethane-bridged PMO materials.

PMO Sample	Size (nm)	Silica Species (%)			Polymerization Degree (%)
		T^1^	T^2^	T^3^	
1	150	2.6	46.6	50.8	82.7
2	200	2.7	48.7	48.6	82.0
3	250	2.7	47.3	50.0	82.5
4	400	2.6	43.8	53.6	83.7
5	600	2.1	44.2	53.2	83.5

**Table 4 t4:** CO_2_ adsorption capacity of different types of silica materials.

No.	Materials		Surface area (m^2^/g)	Temperature (K)	Adsorption Capacity (mmol/g)	Ref.
1	Silica Gel	APTES	340	300	0.41	39
2	MCM-41	/	1490	298	0.66	40
		/	1030	303	0.52	41
		APTES	17	303	0.98	41
3	MCM-48	APTES	1390	298	0.80	42
4	AMS-6	/	921	273	~0.80	43
		APTES	288	273	~2.2	43
6	SBA-15	/	775	318	0.44	44
		APTES	49	318	1.70	44
7	PMO	/	2370	273	1.42	*Present work*
			2370	298	0.97	

Note: the partial pressure for CO_2_ adsorption is 1 bar in all cases.
