# Supplementary material for: Periodic Mesoporous Organosilica Nanocubes with Ultrahigh Surface Areas for Efficient CO2 Adsorption
Source: Sci Rep. 2016 Feb 12;6:20769. doi: 10.1038/srep20769 (PMC4751625; doi:10.1038/srep20769)
Supplement: Supplementary Information [file srep20769-s1.doc]

**Supporting Information**

**Periodic Mesoporous Organosilica Nanocubes with Ultrahigh Surface Areas for Efficient CO_2_ Adsorption**

Yong Wei, ^1^ Xiaomin Li, ^1^ Renyuan Zhang, ^1, 2^ Yong Liu, ^1^ Wenxin Wang, ^1^ Yun Ling, ^1^ Ahmed Mohamed El-Toni, ^3, 4^ & Dongyuan Zhao ^1^

^1^ Department of Chemistry, Collaborative Innovation Center of Chemistry for Energy Materials (*i*ChEM), Laboratory of Advanced Materials, Shanghai Key Laboratory of Molecular Catalysis and Innovative Materials, Fudan University, Shanghai 200433, P. R. China

^2^ School of Materials Science and Engineering, Key Laboratory of Advanced Civil Engineering Materials of Ministry of Education, Tongji University, 4800 Caoan Road, Shanghai, 201804, P. R. China

^3^ King Abdullah Institute for Nanotechnology, King Saud University, Riyadh 11451, Saudi Arabia

^4^ Central Metallurgical Research and Development Institute, CMRDI, Helwan 11421, Cairo, Egypt

Correspondence and requests for materials should be addressed to D. Y. Z ([dyzhao@fudan.edu.cn](mailto:dyzhao@fudan.edu.cn))

E-mail: [dyzhao@fudan.edu.cn](mailto:dyzhao@fudan.edu.cn)

Tel.: +86-21-5163-0205

Fax: +86-21-5163-0307

***Table S1***. **Reactant compositions for the synthesis of ethane-bridged PMO materials from surfactant-templating sol-gel method using tetradecyltrimethylammonium chloride (TTAC) as a template.**

| **Sample** | **TTAC**  **(g)** | **NH_3_•H_2_O**  **(mL)** | **H_2_O**  **(mL)** | **BTSE**  **(mL)** | **Morphology** | **Size**  **(nm)** |
| --- | --- | --- | --- | --- | --- | --- |
| 1 | 0.4 | 1 | 60 | 0.1 | nanocube | 150 |
| 2 | 0.4 | 1.5 | 60 | 0.1 | nanocube | 200 |
| 3 | 0.4 | 2 | 60 | 0.1 | nanocube | 250 |
| 4 | 0.4 | 4 | 60 | 0.1 | nanocube | 400 |
| 5 | 0.2 | 4 | 60 | 0.1 | truncated-cube | 600 |
| 6 | 0.2 | 2 | 60 | 0.1 | truncated-cube | 400 |
| 7 | 0.2 | 1 | 60 | 0.1 | truncated-cube | 300 |

***Table S2*. A series of mesoporous organosilica materials with high surface areas.**

| Sample | Surface Area (m^2^/g) | Reference |
| --- | --- | --- |
| Ethane-bridged PMO | 1390 | 1 |
| Ethane-bridged PMO | 1544 | 2 |
| Ethylene-bridged PMO | 1884 | 3 |

***Table S3***. **Amount (%) of different silica species and condensation degree for the PMO nanocubes with 250 nm in size after the hydrothermal treatment at different temperature.**

| Sample | Silica Species (%) | | | Condensation Degree (%) |
| --- | --- | --- | --- | --- |
|  | T^1^ | T^2^ | T^3^ |  |
| **HT-60** | 2.1 | 40.6 | 57.3 | 85.1 |
| **HT-80** | 2.0 | 35.6 | 62.4 | 86.8 |
| **HT-100** | 0.6 | 30.7 | 68.7 | 89.4 |
| **HT-120** | 0.8 | 30.4 | 68.8 | 89.0 |

***Table S4***. **Physicochemical parameters for the PMO nanocubes with a size of 250 nm after the hydrothermal treatment at different temperature.**

| Sample | Pore Volume (cm^3^/g) | | | |  | Surface Area (m^2^/g) | | | Pore Size  (nm) |  |
| --- | --- | --- | --- | --- | --- | --- | --- | --- | --- | --- |
|  | V_T_ | V_me_ | V _mic_ | V_i_ |  | S_BET_ | S_mic_ | S_me_ |  |  |
| **HT-60** | 1.64 | 0.40 | 0.67 | 0.57 |  | 1500 | 990 | 510 | 3.7/5.7 |  |
| **HT-80** | 1.42 | 0.55 | 0.40 | 0.47 |  | 1120 | 490 | 630 | 3.8/5.7/8.1 |  |
| **HT-100** | 1.38 | 0.88 | 0.12 | 0.38 |  | 940 | 10 | 930 | 3.8/4.9/8.1 |  |
| **HT-120** | 0.89 | 0.58 | 0 | 0.31 |  | 460 | 0 | 460 | 3.8/8.1 |  |


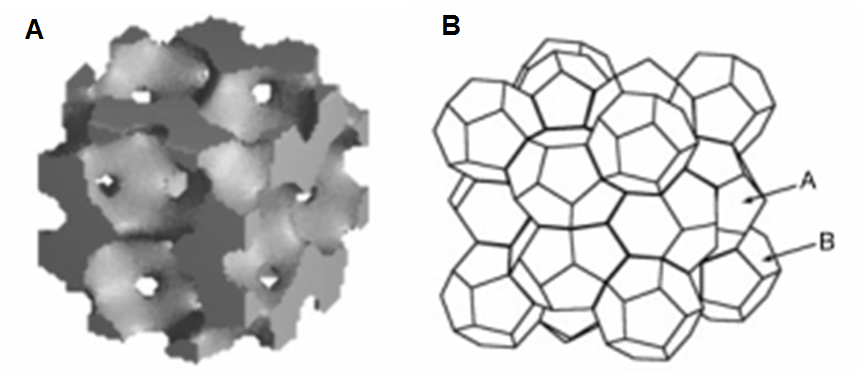


***Figure S1***. 3D structure models of a unit cell (A) and the schematic drawing of the arrangement (B) of A- and B-cages in A_3_B type Pm-3n structures. ^[5]^


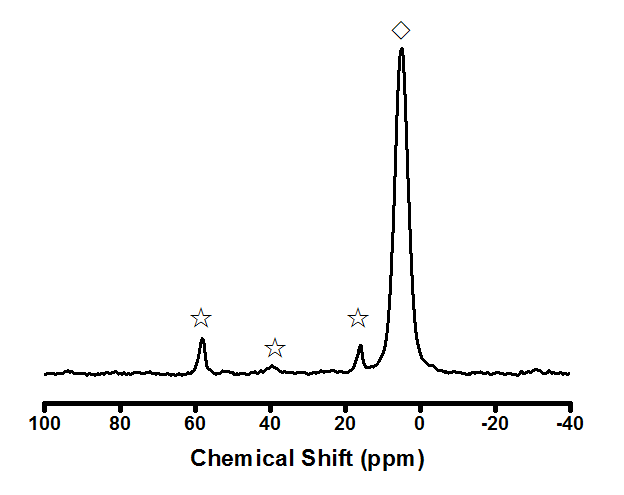


***Figure S2***. ^13^C MAS-NMR spectrum of the PMO nanocubes: the resonance at 4.4 ppm (◇) can be assigned to the C species of the ethane moiety, the bands labelled with an asterisk (☆) are due to the surfactant resi­dues.


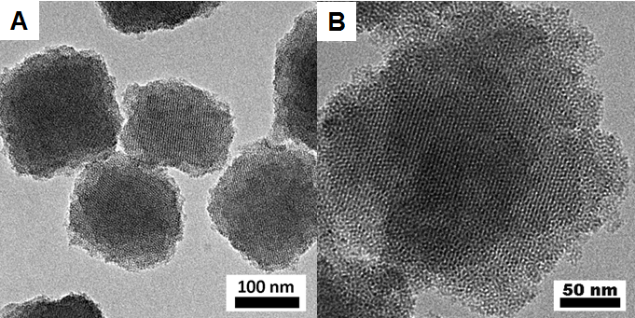


***Figure S3***. TEM images with different magnification of the PMO nanocubes with 150 nm in size prepared from the surfactant-templating sol-gel method at room temperature: (A) low magnification; (b) high magnification.


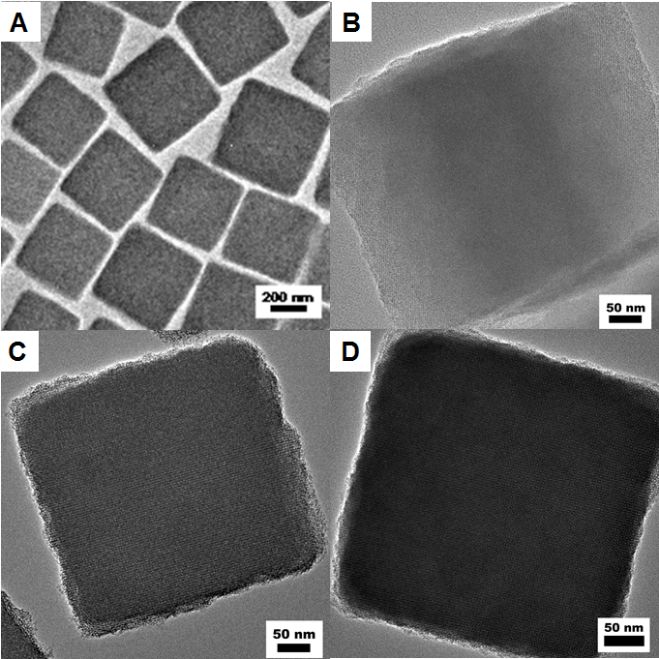


***Figure S4***. TEM images with different magnification of the PMO nanocubes with 400 nm in size.

**
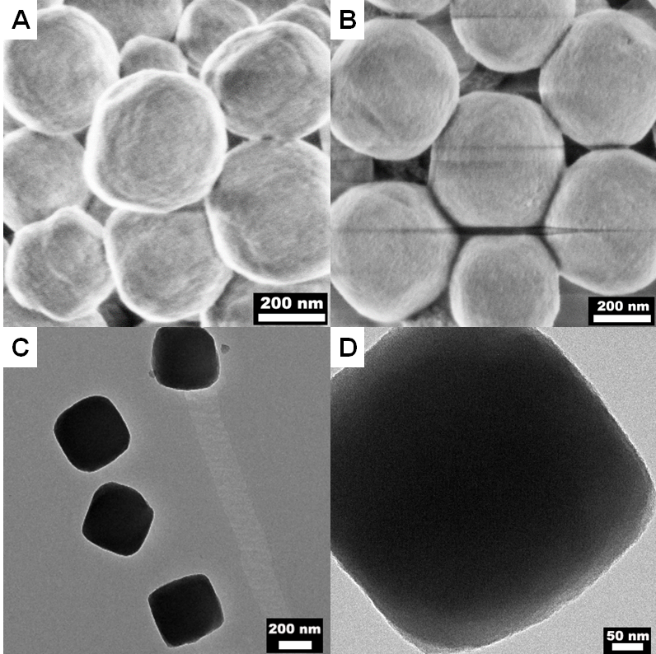
**

***Figure S5***. SEM images (A, B) of the PMO truncated-cubes with a size of 300 nm (A) and 400 nm (B), TEM images (C, D) of PMO truncated-cubes with a size of 400 nm at different magnifications.


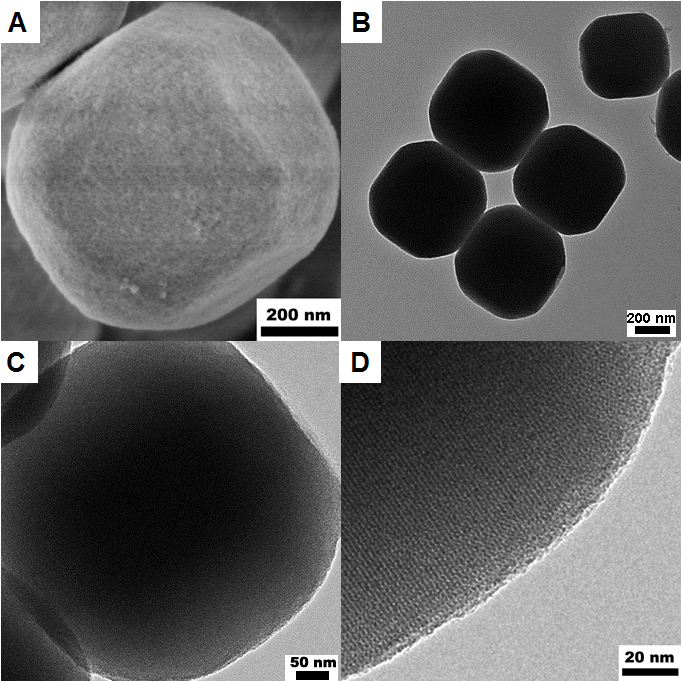


***Figure S6***. HRSEM (A) and HRTEM images at different magnifications (B-D) of the PMO truncated-cubes with 600 nm in size.


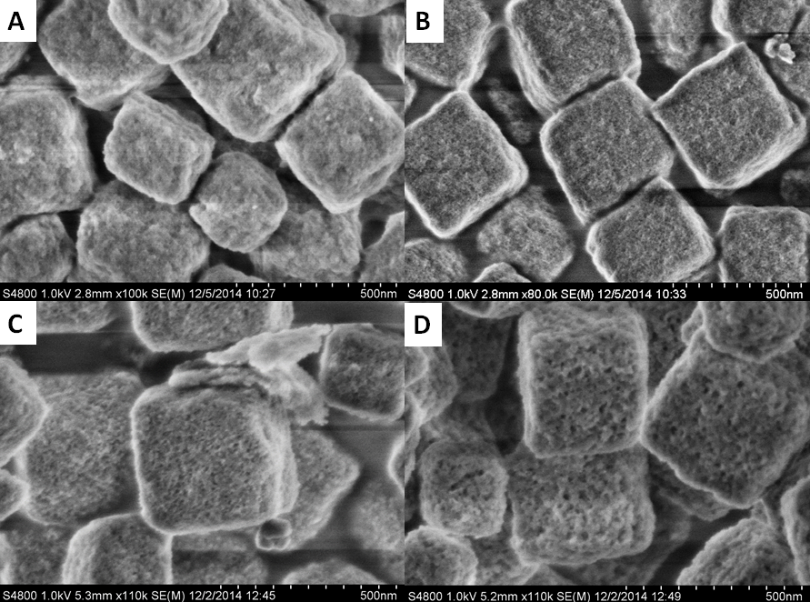


***Figure S7***. SEM images of the PMO nanocubes with the size of 250 nm after hydrothermally treated at different temperatures, (A) 60 °C; (B) 80 °C; (C) 100 °C; (D) 120 °C.


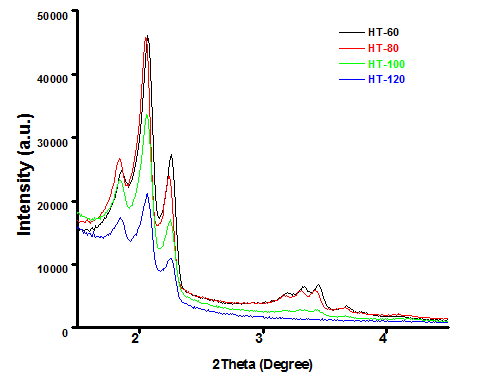


***Figure S8***. Small-angle XRD patterns of the PMO nanocubes with a size of 250 nm after the hydrothermal treated at different temperatures of 60, 80, 100, 120 °C.


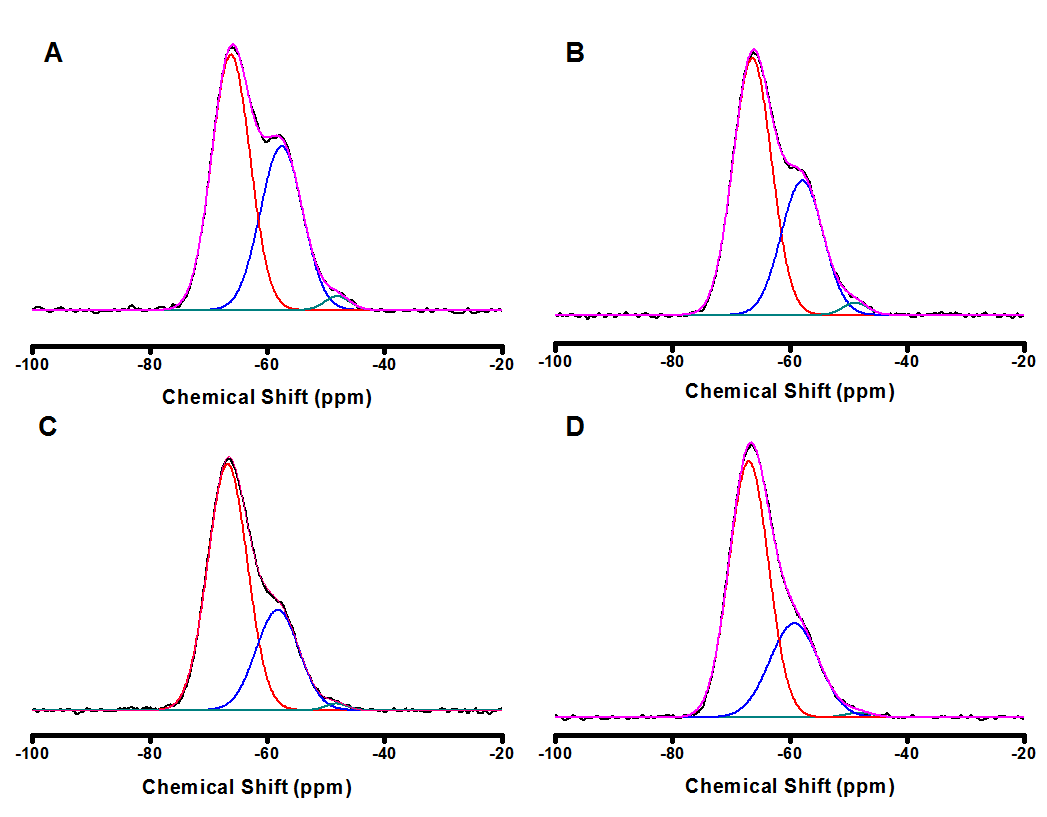


***Figure S9***. ^29^Si MAS-NMR spectra for PMO nanocubes with a size of 250 nm after the hydrothermal treatment at different temperatures: A) 60 °C, B) 80 °C, C) 100 °C, D) 120 °C.


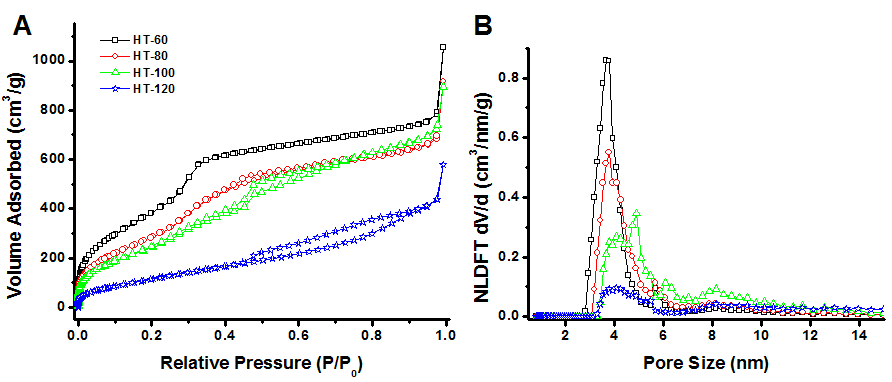


***Figure S10***. Nitrogen adsorption-desorption isotherms (A) and NLDFT pore size distributions (B) of the PMO nanocubes with a size of 250 nm after the hydrothermal treatment at different temperatures.


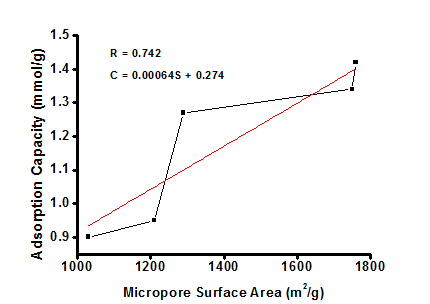


***Figure S11.*** The relationship between CO_2_ capacity and total surface area.


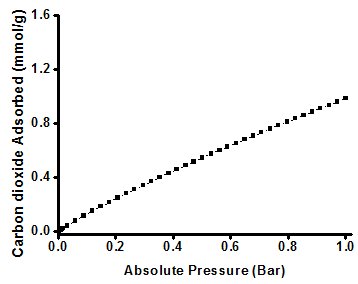


***Figure S12.*** CO_2_ adsorption isotherms at 298 (square) for the PMO nanocubes with a size of 200 nm.


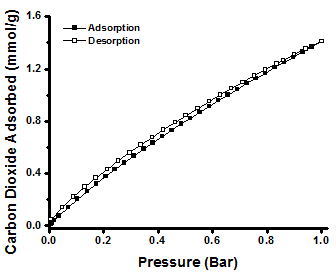


***Figure S13***. CO_2_ adsorption-desorption isotherms at 273 K for the PMO nanocubes with a size of 200 nm.


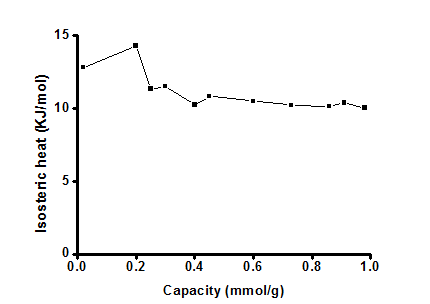


***Figure S14***. Isosteric heat of CO_2_ adsorption on PMO nanocubes with the size of ~ 200 nm.


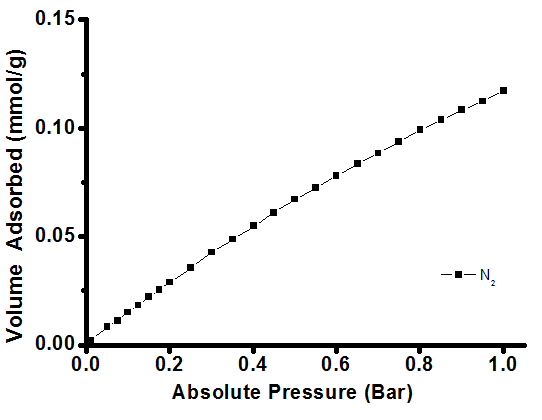


***Figure S15***. N_2_ adsorption isotherms at 273 K for the PMO nanocubes with a size of 200 nm.

***Reference***

1. Mohanty, P., Linn, N. M. K. & Landskron, K. *Langmuir* **26**, 1147–1151 (2010).
2. Yuan, P., Zhao, L. Z., Liu, N., Wei, G. F., Zhang, Y., Wang, Y. H. & Yu C. Z., *Chem. Eur. J.* **15**, 11319 – 11325 (2009).
3. Xia, Y. & Mokaya, R. *Micropor. Mesopor. Mater.* **86**, 231–242 (2005).
4. Sakamoto, Y., Kaneda, M., Terasaki, O., Zhao, D. Y., Kim, J. M., Stucky, G. D., Shin H. J. & Ryoo R. Direct imaging of the pores and cages of three-dimensional mesoporous materials. *Nature* **408**, 449-454 (2000).
